# Repetitive Transcranial Magnetic Stimulation in Spinocerebellar Ataxia: A Pilot Randomized Controlled Trial

**DOI:** 10.3389/fneur.2019.00073

**Published:** 2019-02-12

**Authors:** Brad Manor, Patricia E. Greenstein, Paula Davila-Perez, Seth Wakefield, Junhong Zhou, Alvaro Pascual-Leone

**Affiliations:** ^1^Berenson-Allen Center for Noninvasive Brain Stimulation and Division for Cognitive Neurology, Department of Neurology, Beth Israel Deaconess Medical Center, Boston, MA, United States; ^2^Harvard Medical School, Boston, MA, United States; ^3^Hinda and Arthur Marcus Institute for Aging Research, Hebrew SeniorLife, Roslindale, MA, United States; ^4^Institut Guttman de Neurorehabilitació, Universitat Autonoma de Barcelona, Barcelona, Spain

**Keywords:** rTMS, spinocerebellar ataxia, cerebellum, Standard Ataxia Rating Assessment, standing postural control

## Abstract

Spinocerebellar ataxia (SCA) is a neurodegenerative disorder caused by dysfunction of the cerebellum and its connected neural networks. There is currently no cure for SCA and symptomatic treatment remains limited. We aimed here to examine the effects of a repetitive transcranial magnetic stimulation (rTMS) targeting the cerebellum on clinical impression, postural control and gait in patients with SCA. In this randomized, double-blinded and sham-controlled study, 20 individuals aged 18–75 years with SCA confirmed by genetic testing completed rTMS or sham intervention comprising 20 sessions of MRI-guided stimulation over the cerebellum. Baseline assessments included the Standard Ataxia Rating Assessment (SARA), the 9-hole peg test of manual dexterity, the Timed Up-and-Go (TUG) test, standing postural control with eyes-open and eyes-closed, and gait. Immediate (within 1-week) and 1-month follow-ups were completed. Intervention compliance was high (19 ± 2 of 20 sessions) and no rTMS-related adverse events were reported. rTMS, compared to sham, was associated with greater percent improvement in SARA total score from baseline to the 1-month follow-up (*p* = 0.008). Secondary analyses of individual SARA items revealed that rTMS improved performance within the “stance” sub-score only (*p* = 0.002). This functional change was accompanied by improvement to several objective metrics of postural sway during eyes-open and eyes-closed standing (*p* < 0.008). rTMS did not influence the 9-hole peg test, TUG, or gait kinematics. A 20-session rTMS intervention is safe and feasible for those with SCA. Additional research is warranted to confirm the observed longer-term benefits of this intervention on standing postural control.

**Clinical Trial Registration**: www.ClinicalTrials.gov, identifier: NCT01975909

## Introduction

Autosomal dominant spinocerebellar ataxia (SCA) is associated with degeneration of the cerebellum and its efferent and/or afferent cerebello-thalamocortical tracts (1, 2). Patient with SCA often present with a host of motor symptoms, including deficits to the control of both standing posture and gait (3–5). Such deficits are progressive in nature and greatly increase one's risk of falling (6, 7) and losing functional independence (8). There is currently no cure for SCA and attempts to improve the clinical symptoms of ataxia have been largely unsuccessful and/or short-lasting (9). There is thus an urgent need to develop novel therapeutic interventions for this vulnerable population.

Dysfunction in the cerebellar region and its connected neural networks is thought to be the proximal root cause of movement disorder in patients with SCA (1, 2, 10). Therapeutic strategies aimed at functional improvement of the cerebellum may thus lead to significant clinical benefit within this vulnerable population. Repetitive transcranial magnetic stimulation (rTMS) enables non-invasive modulation of cortical excitability (11). rTMS targeting cerebellar structures is capable of inducing long-lasting changes in the excitability of cerebello-thalamo-cortical pathways (12–14). Shiga et al. (15) reported that as compared to a sham intervention, 21 daily sessions of rTMS targeting the cerebellum improved performance in several short clinical tests of gait and posture, when tested immediately after the intervention was completed, in a cohort of patients with spinocerebellar degeneration. Still, the longer-term effects of rTMS on the clinical impression of symptom severity, as well as the biomechanical control of gait and standing posture, have not been established. We therefore conducted a small, yet well-controlled trial to assess the effects of a four-week, 20-session rTMS intervention targeting the cerebellum, as guided by individual brain anatomy using structural MRI, on the clinical severity of SCA and the control of standing posture and gait using quantitative kinematic assessments, in patients with SCA as confirmed by genetic testing.

## Methods

### Trial Design

A parallel-group, randomized controlled trial was conducted (NCT01975909). Enrolled participants completed baseline assessments and a structural brain MRI. They were then assigned to receive the rTMS or sham intervention via permuted block randomization with stratification by sex. rTMS was administered by study personnel uninvolved in other study procedures. Participants and the study staff who assessed outcomes were blinded to intervention arm. Immediate (i.e., within 1 week of intervention completion) and 1-month follow-up assessments were completed.

### Trial Registration

This study was registered prospectively at https://clinicaltrials.gov/ (NCT01975909).

### Participants

Participants were recruited between 2013 and 2015 from the Neurogenetics Clinic and Movement Disorders Center at the Beth Israel Deaconess Medical Center (BIDMC), local ataxia support organizations, the National Ataxia Foundation and clinicaltrials.gov.

Inclusion criteria included SCA confirmed by genetic testing, age 18–75, the ability to ambulate without assistance from another person (canes/walkers allowed), a score >3 on the “gait” subsection of the Scale for the Assessment and Rating of Ataxia (SARA) (16), a negative pregnancy test and stable medications. Exclusion criteria were unstable neurological illness or concomitant medical condition (i.e., stroke, arthritis, etc.), clinically-significant abnormalities on screening (e.g., basic lab work or EKG abnormalities), concurrent participation in another clinical study, history of substance abuse, untreated depression, dementia, psychiatric illness, subjects who were wheelchair bound, Mini Mental Status Exam score <24, legal incapacity or limited legal capacity. TMS and MRI-specific exclusions included metal in the head, history of neurosurgical procedures, ferromagnetic bioimplants, metallic paint, history of seizure disorder, claustrophobia, current usage of buproprion or other medications that may increase risk of TMS-induced seizures.

**Figure 1:**
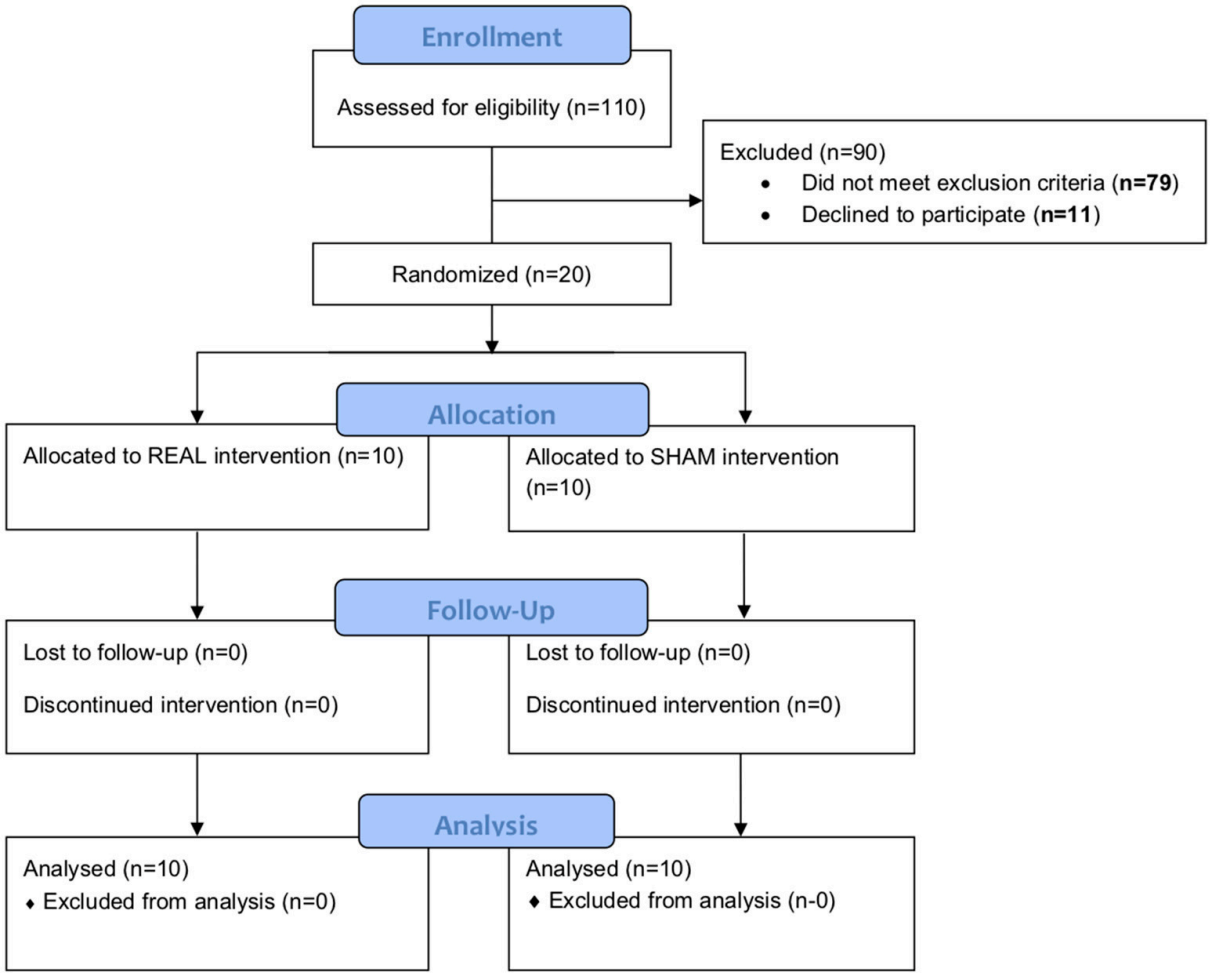
Study CONSORT diagram. One hundred and ten individuals were screened. Seventy-nine were ineligible and 11 were uninterested. The remaining 20 completed baseline testing. Ten were randomized to the rTMS intervention and ten to sham. All of them completed the intervention and both follow-ups.

We screened 110 individuals. Seventy-nine were ineligible and 11 were uninterested (Figure 1). The remaining 20 completed baseline testing. Ten were randomized to the rTMS intervention (women = 8; SCA type 3 = 8; mean ± SD age = 53 ± 9 years; height = 164 ± 10 cm; body mass = 71 ± 13 kg) and ten to sham (women = 8; SCA type 3 = 6; age = 49 ± 4 years; height = 161 ± 6 cm; body mass = 67 ± 13 kg). All 20 participants were naïve to TMS and completed the intervention and all study assessments.

### Intervention

The rTMS intervention comprised 20 sessions over 4 consecutive weeks. A Magstim 200 (UK) and 14 cm circular coil delivered stimuli at 100% of maximal stimulator output intensity with the coil centered over three regions: the inion, 4 cm lateral to the left of the inion, 4 cm lateral to the right of the inion. Participants were asked to lay their head down on a pillow placed on a table in front of them, and the handle of the TMS coil was held facing upwards. Structural MRIs were used to locate and mark each region for rTMS and neuronavigation using Brainsight® (Rogue Resolutions, Cardiff, Wales) ensured that all stimuli for a given region within and across daily sessions targeted the same cerebellar regions. For each region, five pulses separated by 6 s were delivered counter-clockwise, followed by five pulses delivered clockwise, for a total of 10 pulses per region and session, and a total of 30 pulses per session (15). For sham intervention, the same parameters and procedures were used except the coil was angled 90 degrees from the scalp, inducing non-measurable changes of the excitability in cerebellum (17).

### Ethics

This study was carried out in accordance with the recommendations of the BIDMC Institutional Review Board with written informed consent from all subjects. All participants gave written informed consent in accordance with the Declaration of Helsinki. The protocol was approved by the BIDMC Institutional Review Board.

### Assessments

Assessments were conducted at the Harvard-Catalyst Clinical Research Center at the BIDMC at approximately the same time of day. Screening included health history, neurological exam, the Mini Mental State Examination and the SARA. A nurse recorded medications, resting EKG, vital signs, height and body mass. Hematology, pregnancy testing (if applicable), renal and liver panels were completed. A study physician reviewed screening data to determine eligibility. The SARA, nine-hole peg test, Timed Up-and-Go (TUG) and biomechanical assessments of standing posture and gait were measured at baseline and at both follow-ups. Assistive devices were allowed on all tests except standing posture.

### Scale for the Assessment and Rating of Ataxia (SARA)

The clinical severity of SCA was measured using the valid and reliable SARA scoring scale (16). This scale consists of eight items related to gait, stance, sitting, speech, finger-chase test, nose-finger test, fast alternating movements, and heel-shin test.

### 9-Hole Peg Test

The peg test was used to assess fine motor control hand and manual dexterity (18). Participants were asked to remove the pegs from the holes, one by one, and then replace them back into the container. The time to complete the test was recorded, with longer times reflecting worse performance.

### Timed Up-and-Go Test (TUG)

The TUG test was used to assess functional mobility (19). The time taken to stand from a chair, walk forward three meters, turn around, walk back, and return to a seated position was recorded.

### Assessment of Standing Posture and Gait

Standing postural control and gait were assessed using standard procedures. Postural control was assessed by measuring postural sway (i.e., center of pressure) fluctuations (240 Hz) during standing on a stationary force platform (AMTI, Watertown, MA). Participants were asked to stand barefoot on the platform and complete two 30-s trials under both eyes-open and eyes-closed conditions. Trial order was randomized. Tissue paper was placed on the force platform and foot placement of each participant was outlined prior to the first trial. This outline was then used throughout all future assessments and trials to ensure consistent foot placement over time. Participants were instructed to “stand as still as possible” prior to each trial. For eyes-open trials, participants were further instructed to visually focus on a target “X” placed on the wall approximately 3-m in front of them at eye-level.

Gait was assessed by measuring the kinematics of walking using the wireless Mobility Lab® system (APDM, Seattle WA) during a 90-s walk. Participants were instructed to walk at their normal, preferred paced. The use of assistive device (e.g., cane) was allowed and if it was used, the same device was used throughout the study.

### Study Outcomes

The primary outcome of this study was the SARA total score. Secondary outcomes included performance within clinical functional tests (9-hole peg test, TUG) and metrics related to standing postural control and gait. Standing postural control metrics were chosen based upon their sensitivity to change in SCA severity (3) and included average sway speed (i.e., center-of-pressure path length divided by trial duration) and area (i.e., the area of a confidence ellipse enclosing 95% of the center-of-pressure trajectory) during both eyes-open and eyes-closed conditions. Gait metrics included average walking speed, stride time variability (i.e., the coefficient of variation about the mean between consecutive heel strikes of the right foot) and double support time (i.e., the average percentage of each stride time spent with both feet on ground), as each has been linked to SCA severity and related functional decline (4, 5).

### Statistical Analysis

Analyses were performed using JMP Pro 12 (SAS Institute, Cary, NC). Descriptive statistics summarized group demographics and outcomes. Potential between-group differences in baseline characteristics were tested with Student's *t*-tests or chi-square tests. Primary analyses examined the effects of rTMS on primary and secondary outcomes using two-way repeated-measures ANOVAs. As groups differed in several measures of functional performance at baseline, dependent variables were *the percent change in each outcome from baseline to each follow-up visit*. Model effects included follow-up time (within-subject: one-week, one-month), group (between-subject: rTMS, sham), and their interaction. Models were completed with and without adjustments for age, sex, and intervention compliance. Two secondary analyses were complete based upon the observed effects of rTMS intervention on SARA performance. First, as a potential placebo effect was observed in SARA total score at the one-week follow-up, a one-way repeated-measures ANOVA was completed to determine the between-subject effect of group on the percent change in SARA total score *only* from baseline to the 1-month follow-up visit. Second, similar two-way models as described above were completed to examine the effects of intervention on each of the nine SARA items. Significance level for all statistical tests within this pilot study was set to *p* < 0.05. Effect sizes of significant models was measured using Cohen's d and the partial eta square metric (η^2^).

*Sample size considerations*: To our knowledge, this was the first pilot study to systematically test the effects of MRI-guided cerebellar rTMS on the SARA and other functional and biomechanics outcomes over a 1-month follow-up period in patients with genetically-confirmed SCA. While the primary objective of this study was to provide the data needed to appropriately power more definitive trials in the future, we conducted a priori sample size calculations based upon Shiga et al. (15). That study reported the immediate after-effects of a non-MRI-guided, 21-day cerebellar rTMS intervention on 10-m walking speed in 74 patients with suspected SCA. The rTMS group decreased their 10-m walk time (from 14.3 ± 1.8 to 9.9 ± 0.7 s, mean ± SD) significantly more than those receiving the sham treatment (from 13.7 ± 1.2 to 13.6 ± 1.2 s). We estimated that a sample size of 20 would provide over 80% power to detect a similar effect size, after adjusting for three covariates.

## Results

The demographic, SCA and health characteristics of each participant are listed in Table 1. The groups receiving rTMS and sham intervention had similar age, height and body mass. At baseline, the rTMS group, as compared to the sham group, exhibited lower SARA scores (*p* = 0.01), faster 9-hole peg test time (*p* = 0.03) and faster postural sway speed during eyes-open standing (*p* = 0.01) (Table 2). No other between-group baseline differences were observed. Baseline functional outcomes did not differ by sex (*p* > 0.56) and were not significantly correlated with participant age (*p* > 0.33) or BMI (*p* > 0.40). Intervention compliance was high (19 ± 2 of 20 sessions) and similar between groups. The rTMS and sham interventions were well-tolerated and no unexpected side effects or adverse events were reported.

**Table 1:** Demographics and baseline functional performance of each participant.

**Participant ID**	**Age (years)**	**Sex**	**BMI**	**SCA-type**	**rTMS**	**SARA**	**9-hole peg test (s)**	**TUG time (s)**
P001	61	Male	29.3	3	Real	22.5	78.0	65.0
P002	52	Female	21.7	3	Sham	13.5	39.5	22.1
P003	45	Female	25.8	3	Sham	24	45.8	68.8
P004	45	Female	21.9	3	Sham	15.5	51.7	49.7
P005	47	Male	27.1	1	Sham	13.5	41.8	20.4
P006	38	Male	19.8	3	Real	10	34.8	42.6
P007	47	Male	24.5	3	Sham	24.5	76.9	119.9
P008	52	Male	27.3	3	Real	14	37.2	24.4
P009	44	Female	28.3	3	Real	11	32.4	21.2
P010	54	Female	18.1	6	Sham	19.5	61.0	51.3
P011	65	Female	30.2	3	Real	14.5	40.5	39.3
P012	47	Female	29.3	2	Sham	19	55.2	33.5
P013	54	Female	28.7	3	Real	16.5	28.6	36.0
P014	56	Female	32.9	8	Sham	12	29.9	31.3
P015	46	Female	22.5	3	Sham	16	37.9	35.3
P016	49	Female	20.1	3	Real	15	30.4	24.9
P017	49	Female	32.3	14	Sham	13.5	18.3	20.6
P018	50	Male	25.6	6	Real	13	27.7	18.8
P019	47	Female	22.9	3	Real	11	21.1	14.3
P020	68	Female	25.6	6	Real	18.5	32.4	18.8

**Table 2:** SCA severity, postural control and gait outcomes (mean ± SD) at baseline and follow-up.

	**rTMS**			**Sham**		
	**Baseline**	**Follow up (immediate)**	**Follow-up (1 month)**	**Baseline**	**Follow up (immediate)**	**Follow-up(1 month)**
SARA (total)	13.7 ± 2.8	10.7 ± 3.4	9.8 ± 2.6	17.1 ± 4.5	12.9 ± 4.9	14.7 ± 4.0
TUG	26.7 ± 10.1	22.5 ± 7.8	20.2 ± 5.6	32.0 ± 16.6	30.5 ± 11.6	31.5 ± 13.9
9-hole peg test (sec)	31.6 ± 5.7	30.6 ± 5.6	30.8 ± 5.4	42.3 ± 13.1	42.3 ± 14.3	41.0 ± 13.5
**POSTURAL SWAY**						
**Eyes-open**						
Speed (mm/s)	41.2 ± 15.9	27.0 ± 10.2	24.8 ± 10.7	21.5 ± 9.0	20.3 ± 8.9	22.9 ± 7.1
Area (mm^2^)	639 ± 376	467 ± 228	436 ± 173	602 ± 636	479 ± 303	674 ± 249
**Eyes-closed**						
Speed (mm/s)	81.4 ± 46.5	51.0 ± 24.3	55.0 ± 30.0	61.0 ± 17.1	63.0 ± 40.4	68.4 ± 40.3
Area (mm^2^)	1992 ± 1337	824 ± 404	1303 ± 839	868 ± 780	1156 ± 938	1517 ± 1382
**GAIT**						
Speed (m/s)	1.0 ± 0.3	1.0 ± 0.4	1.0 ± 0.4	0.9 ± 0.4	0.9 ± 0.4	0.9 ± 0.4
Variability (%)	7.0 ± 3.3	5.9 ± 2.3	5.6 ± 3.8	8.0 ± 5.8	7.8 ± 6.2	8.3 ± 5.9
Double support (%)	27.9 ± 6.9	25.2 ± 7.8	24.2 ± 11.3	27.6 ± 15.2	30.0 ± 14.2	31.2 ± 14.5

*SCA, spinocerebellar ataxia; rTMS, repetitive transcranial direct current stimulation; SARA, scale for the assessment and rating of ataxia; TUG, Timed Up-and-Go*.

**Figure 2:**
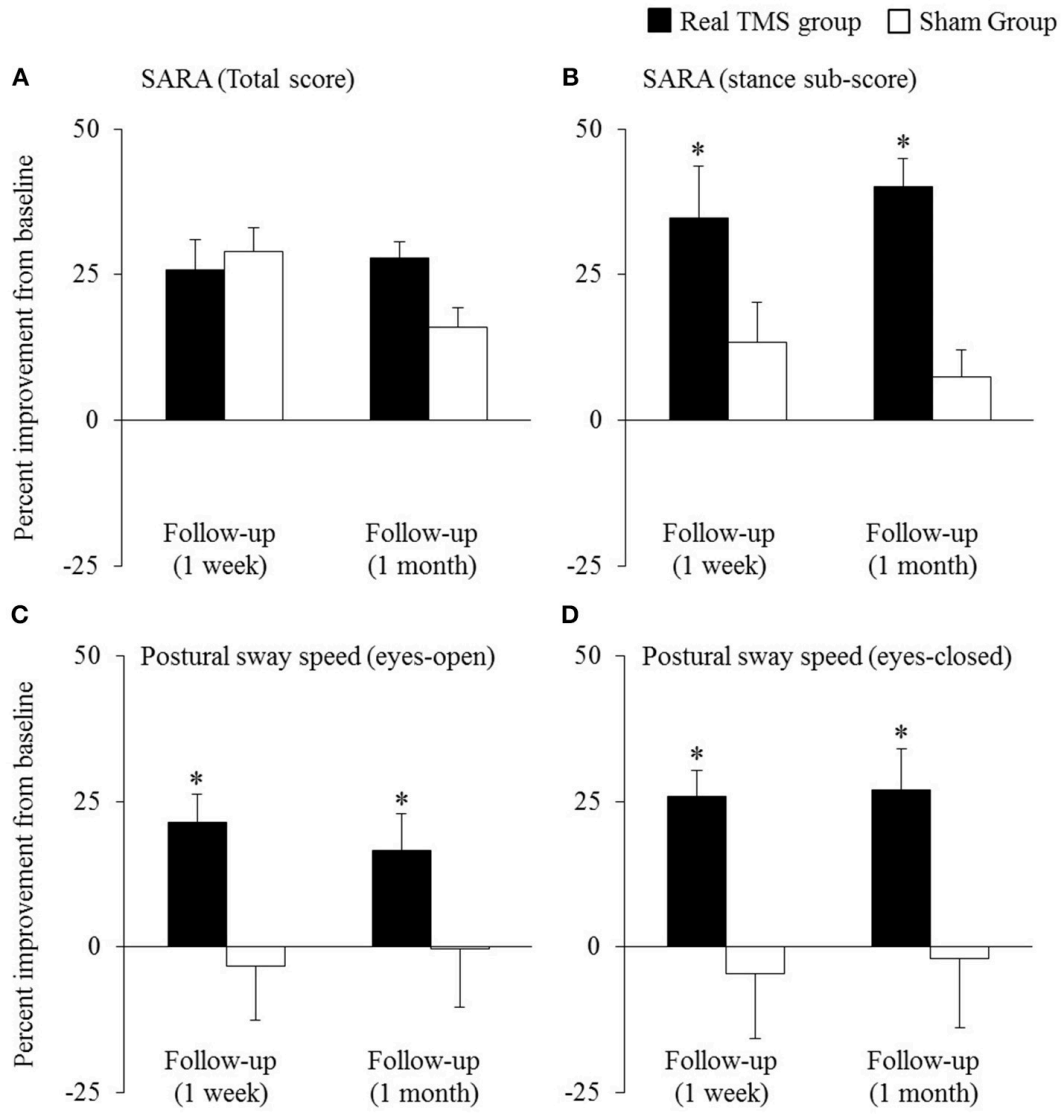
The effects of cerebellar rTMS on the clinical assessment of ataxia and standing postural control (mean ± SE). Both groups exhibited relatively-large improvement (i.e., percent reduction) in SARA total score from baseline to the immediate follow-up **(A)**. The rTMS group, however, exhibited greater percent improvement in this outcome, as compared to the sham group, at the 1-month follow-up. Secondary analyses revealed that compared to sham, the rTMS group exhibited greater improvements specifically within the SARA “stance” sub-score **(B)**, yet no other SARA item (outcomes not pictured). Moreover, rTMS improved postural sway speed (i.e., sway speed decreased) during both eyes-open **(C)** and eyes-closed **(D)** standing. ^*^Indicates significant main effects of group within two-way, repeated-measures ANCOVAs adjusted for age, sex, and intervention compliance.

Primary and secondary outcomes are presented by intervention group in Table 2. A two-way, repeated-measures ANOVA revealed a trend toward a main effect of group for SARA total score (*F* = 2.0, *p* = 0.16, Cohen's d = 0.5, η^2^ = 0.06). As can be observed in Figure 2A, both groups exhibited relatively large percent reductions (i.e., improvements) in this outcome from baseline to the immediate follow-up. Secondary analyses omitting the one-week follow-up assessment revealed that the rTMS intervention, as compared to sham, induced a greater percent decrease in SARA total scores from baseline to the 1-month follow-up (*F* = 9.3, *p* = 0.008, Cohen's d = 1.3, η^2^ = 0.38) (Figure 2A). This effect was independent of age, sex, and intervention compliance. Spurred by this observation, the effects of intervention on the percent change from baseline to the 1-month follow-up in each of the nine SARA sub-scores were also examined. rTMS, compared to sham, improved performance within the “stance” sub-score (*F* = 10.4, *p* = 0.002, Cohen's d = 0.9; η^2^ = 0.24) (Figure 2B). No other item-specific changes within the SARA exam were observed between groups.

The beneficial effect of rTMS on the clinical assessment of posture was corroborated by improvements within several objective kinematic metrics of standing postural sway. As compared to sham, those who completed the rTMS intervention exhibited a greater percent decrease in postural sway speed when standing with eyes open (group effect: *F* = 9.5, *p* = 0.004, Cohen's d = 1.0; η^2^ = 0.28) and eyes closed (group effect: *F* = 11.4, *p* = 0.002, Cohen's d = 1.0, η^2^ = 0.26) (Figures 2C,D). rTMS, as compared to sham, also reduced sway area during eyes-closed standing (*F* = 8.5, *p* = 0.007, Cohen's d = 0.9, η^2^ = 0.17). Each of these observed group effects were independent of age, sex, and intervention compliance. No main effects of time, nor group by time interactions, were observed for any metric.

**Figure 3:**
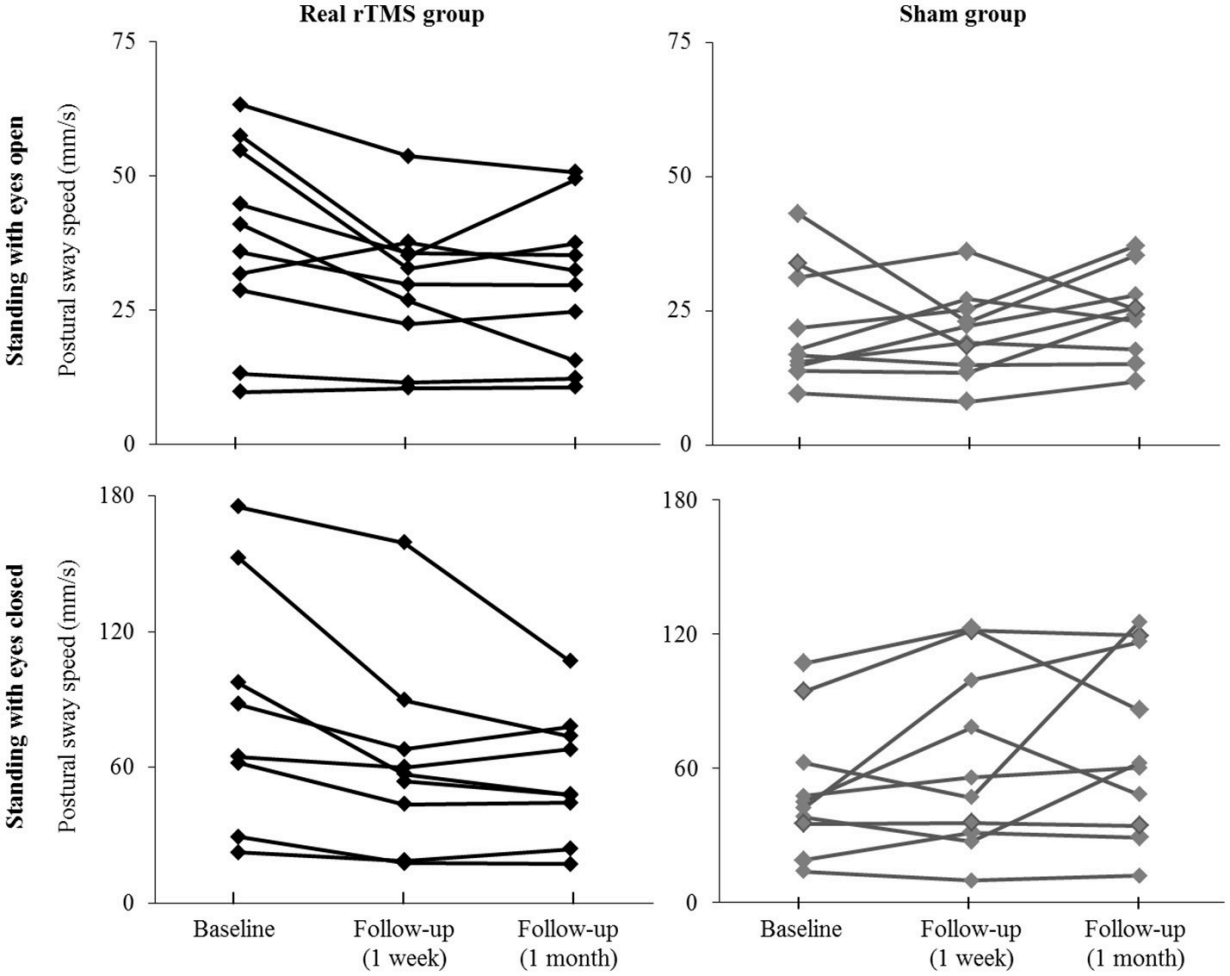
Individual effects of cerebellar rTMS intervention on standing postural control. The average speed of standing postural sway fluctuations for each participant when standing with eyes open **(top)** and eyes closed **(bottom)** at baseline and at the immediate and 1-month follow-up assessments. Two participants with extremely high sway speed values at baseline (out of the mean ± 2XS.D.) were excluded from the figure for better visual effects.

Participant-level results of intervention on postural sway speed are presented in Figure 3. Seven of ten participants who received rTMS exhibited slower sway speed (i.e., better standing postural control) during eyes-open standing at the 1-month follow-up assessment as compared to baseline. In contrast, only two sham participants demonstrated such improvements. Nine rTMS participants exhibited slower sway speed during eyes-closed standing 1 month following the intervention, as compared to only three sham participants.

rTMS did not have significant effects on performance within the 9-hole peg test, the TUG test of mobility, or on metrics of gait performance (i.e., walking speed, stride time variability, double support time).

## Discussion

This randomized sham-controlled pilot clinical trial provided preliminary evidence that cerebellar rTMS intervention is safe and feasible for patients with SCA who vary considerably in age and disease progression. Participants who completed the 4-week rTMS intervention exhibited significant improvements to both clinical and kinematic outcomes of standing postural control, over a 1-month follow-up period, as compared to those who received the sham intervention. rTMS did not influence manual dexterity, functional mobility or gait kinematics during the follow-up period. Still, the potential benefits of rTMS on posture warrant larger, more definitive trials to establish the effects of MRI-guided cerebellar rTMS on postural control, as well as activities of daily living and quality of life in SCA.

rTMS intervention improved the clinical impression of standing posture (i.e., the SARA “stance” sub-score), along with multiple metrics of postural sway when standing with eyes open and closed. In each case, the value of Cohen's d was greater than 0.8, indicating a large effect size of intervention on this important functional domain. These results are supported by Shiga et al. (15), who reported that an rTMS intervention targeting the cerebellum improved the capacity to stand with different bases of support (i.e., foot placements), when tested soon after completion of the intervention. The current results further suggest that rTMS may enhance standing balance by improving the capacity to control (i.e., minimize) the speed and magnitude of postural sway, and that such improvements may persist for at least 1 month. Future large-scale studies are thus needed to confirm these results, and, to establish the mechanisms through which cerebellar stimulation may improve standing posture. The regulation of posture when standing relies upon a complex control system that utilizes and integrates multiple sources of sensory input within spinal and supra-spinal networks to generate both automatic and volitional corrective muscular actions (20–22). Neuroimaging studies indicate that the task of standing, as compared to sitting, activates a distributed network of brain regions including the cerebellum, primary motor cortex, and other regions involved in sensory integration and/or cognitive-motor control (22, 23). SCA causes postural disturbances, at least in part, by impairing functional activation of cerebellar Purkinje cells. This in turn inhibits cortical motor activation via a complex neural pathway involving the dentate nucleus (10). Several recent studies have demonstrated that cerebellar rTMS is capable of facilitating motor cortical activation via modulation of Purkinje cell excitability (24–26). Future studies employing paired-pulse TMS methodology are thus needed to examine the effects of cerebellar rTMS intervention on motor cortex excitability, and its links to postural control, in those individuals with SCA.

Both the rTMS and sham groups demonstrated improved performance in the SARA total score at the immediate follow-up assessment, as compared to baseline (Figure 2A). However, within the sham group, this initial improvement was attenuated at the 1-month follow-up assessment. This short-lasting positive effect of rTMS in the sham group was only present for the SARA total score, and may have reflected a placebo effect of the intervention. Such placebo effects have been reported in several other studies in those with cerebellar degeneration (27, 28). For example, in a multi-center study of patients with cerebellar ataxia, ondansetron and placebo interventions resulted in a similar improvement in performance on the International Cooperative Ataxia Rating Scale (ICARS) over the follow-up period (27). Additionally, in the current study, a proportion of participants within each intervention arm stayed within the hospital's Clinical Research Center for the duration of the study and thus received ongoing clinical research staff supervision that was likely greater than their normal care. The possibility that this increased attention confounded the initial effects of intervention, together with the potential for a significant placebo effect within this population, are thus important factors to consider when designing future trials of rTMS or other therapies in SCA.

While the effects of rTMS on outcomes related to postural control were independent of age, sex, and intervention compliance, the small sample size of this pilot study limited our capacity to statistically control for additional, important covariates such as SCA subtype. Considerable between-subject variance in symptom severity and functional status was present, and despite random assignment to intervention arm, the rTMS group exhibited better functional performance at baseline. Nevertheless, the observations that the rTMS intervention was well-attended and not associated with any unexpected side effects or adverse events, together with preliminary evidence of improved postural control, highlight the potential for this form of non-invasive brain stimulation to serve as a therapeutic rehabilitative strategy for SCA.

## Author Contributions

BM, PG, and AP-L designed the study. BM, PG, SW, and PD-P collected the data. BM, PD-P, and JZ analyzed the data and performed statistical analyses. BM, PG, PD-P, JZ, and AP-L drafted the manuscript. All authors contributed to and approved the final version.

### Conflict of Interest Statement

AP-L serves on the scientific advisory boards for Nexstim, Neuronix, Starlab Neuroscience, Neuroelectrics, Axilum Robotics, Magstim Inc., and Neosync; and is listed as an inventor on several issued and pending patents on the real-time integration of transcranial magnetic stimulation with electroencephalography and magnetic resonance imaging. The remaining authors declare that the research was conducted in the absence of any commercial or financial relationships that could be construed as a potential conflict of interest.
